# The association between intraoperative hyperglycemia and cerebrovascular markers

**DOI:** 10.7150/ijms.51364

**Published:** 2021-01-21

**Authors:** Cornelia Knaak, Ilse MJ Kant, Florian Lammers-Lietz, Claudia Spies, Theo D Witkamp, Georg Winterer, Gunnar Lachmann, Jeroen de Bresser

**Affiliations:** 1 Department of Anesthesiology and Operative Intensive Care Medicine (CCM, CVK), Charité - Universitätsmedizin Berlin, corporate member of Freie Universität Berlin, Humboldt-Universität zu Berlin, and Berlin Institute of Health, Augustenburger Platz 1, D-13353 Berlin, Germany.; 2 Department of Radiology, University Medical Center Utrecht, Utrecht, The Netherlands.; 3 Department of Intensive Care Medicine and Brain Center Rudolf Magnus, UMC Utrecht, Utrecht, The Netherlands.; 4 Pharmaimage Biomarker Solutions GmbH, Robert-Rössle-Str. 10, D-13125 Berlin, Germany.; 5 Berlin Institute of Health (BIH), Anna-Louisa-Karsch-Str. 2, D-10178 Berlin, Germany.; 6 Department of Radiology, Leiden University Medical Center, Leiden, The Netherlands.

**Keywords:** Intraoperative hyperglycemia, Neuroimaging MRI, perfusion, cerebral small vessel disease, vascular lesions.

## Abstract

**BACKGROUND AND PURPOSE:** Hyperglycemia can lead to an increased rate of apoptosis of microglial cells and to damaged neurons. The relation between hyperglycemia and cerebrovascular markers on MRI is unknown. Our aim was to study the association between intraoperative hyperglycemia and cerebrovascular markers.

**METHODS:** In this further analysis of a subgroup investigation of the BIOCOG study, 65 older non-demented patients (median 72 years) were studied who underwent elective surgery of ≥ 60 minutes. Intraoperative blood glucose maximum was determined retrospectively in each patient. In these patients, preoperatively and at 3 months follow-up a MRI scan was performed and white matter hyperintensity (WMH) volume and shape, infarcts, and perfusion parameters were determined. Multivariable logistic regression analyses were performed to determine associations between preoperative cerebrovascular markers and occurrence of intraoperative hyperglycemia. Linear regression analyses were performed to assess the relation between intraoperative hyperglycemia and pre- to postoperative changes in WMH volume. Associations between intraoperative hyperglycemia and postoperative WMH volume at 3 months follow-up were also assessed by linear regression analyses.

**RESULTS:** Eighteen patients showed intraoperative hyperglycemia (glucose maximum ≥ 150 mg/dL). A preoperative more smooth shape of periventricular and confluent WMH was related to the occurrence of intraoperative hyperglycemia [convexity: OR 33.318 (95 % CI (1.002 - 1107.950); p = 0.050]. Other preoperative cerebrovascular markers were not related to the occurrence of intraoperative hyperglycemia. Intraoperative hyperglycemia showed no relation with pre- to postoperative changes in WMH volume nor with postoperative WMH volume at 3 months follow-up.

**CONCLUSIONS:** We found that a preoperative more smooth shape of periventricular and confluent WMH was related to the occurrence of intraoperative hyperglycemia. These findings may suggest that a similar underlying mechanism leads to a certain pattern of vascular brain abnormalities and an increased risk of hyperglycemia.

## Introduction

Hyperglycemia occurs frequently during surgery and has been related to poor clinical outcome such as acute kidney injury, cognitive impairment and increased rates of wound infection resulting from compromised immune functioning 1-4. Glycemic control in critically ill patients reduced postoperative complications and improved survival, particularly in septic patients 5. Prevention of hyperglycemia is critical to neuroprotection as microglial activation, loss of astrocytes and neuronal damage in the hippocampus and frontal cortex were more prevalent in hyperglycemic than in normoglycemic critically ill patients 6. Furthermore, microglial apoptosis was found in patients who died from a septic shock and had suffered from hyperglycemia during their intensive care unit stay 7.

As changes in brain structures may develop before cognitive decline becomes noticeable, detection of markers indicating neuronal damage is of particular interest. Studies in older individuals in the general population using magnetic resonance imaging (MRI) showed an association between hyperglycemia and a larger number of brain infarcts and more white matter hyperintensities (WMH) 8. Furthermore, patients with diabetes may be more prone to develop WMH 9-13.

Studies investigating the relation between intraoperative hyperglycemia and cerebral markers on MRI are scarce. In our previous study, we found no association between intraoperative hyperglycemia and pre- to postoperative volume changes of the hippocampus and the hippocampal subfields, the frontal lobe, and frontal cortical thickness 14. To the best of our knowledge no previous studies have focused on the association between intraoperative hyperglycemia and cerebrovascular markers. Therefore, our aim was to study the association between intraoperative hyperglycemia and cerebrovascular markers 15.

## Methods

### Study participants and groups

This is a further subgroup analysis of a previously published cohort 15 that is a subgroup investigation of the BIOCOG study (Biomarker Development for Postoperative Cognitive Impairment in the Elderly) 16. Patients with at least one intraoperative blood glucose measurement and MRI scans before and 3 months after surgery were included. Blood glucose levels were assessed intraoperatively at the discretion of the anesthesiologist and for the purpose of this study, abstracted from patient charts. The BIOCOG study is a prospective, multi-center observational study for biomarker development for risk prediction of cognitive impairment in elderly surgical patients (EA2/092/14, NCT02265263, www.biocog.eu). Patients were eligible for BIOCOG if they were at least 65 years of age, were scheduled for elective surgery with an expected duration of surgery of at least 60 minutes and had a baseline Mini Mental Status Examination (MMSE) of at least 24 points. For this subgroup investigation, preoperative MRI scans and postoperative MRI scans at 3 months follow-up and at least one blood glucose measurement during surgery were considered additional inclusion criteria. The study was approved by the ethics committee of Charité - Universitätsmedizin Berlin and written informed consent was obtained from all patients. The study was conducted in accordance with the criteria set by the ICH-GCP and the declaration of Helsinki.

### Measurement of pre- and perioperative parameters

Baseline demographic data and medical parameters including preoperative MMSE, prior history of diabetes, preoperative blood glucose levels and HbA1c were assessed before surgery. Blood samples were obtained preoperatively to determine fasting blood glucose levels. Perioperative parameters (i.e. type of surgery and surgical time as well as intraoperative blood glucose measurements) were retrospectively recorded by chart review. Postoperative measures including length of intensive care unit stay, total length of hospital stay, and incidence of postoperative infections were documented. Intraoperative glucose minimum, maximum and mean were determined. As maintenance of a blood glucose level < 150 mg/dL is recommended in septic patients, where blood glucose levels above 150 mg/dL have been associated with cerebral damage, we defined hyperglycemia by at least one blood glucose measurement ≥ 150 mg/dL 6, 17. Evidence for postoperative infections was retrospectively assessed by chart review until day 7 after surgery.

### MRI measurement of cerebrovascular markers

#### MRI scans

Participants were scanned on a 3T Magnetom TrioTim (Siemens Healthcare, Erlangen, Germany) MRI scanner. The MRI scanning protocol comprised a 3-dimensional T1-weighted sequence (repetition time [TR]/echo time [TE] 2500/4.77 ms, voxel-size = 1.0 x 1.0 x 1.0 mm^3^), a 3D susceptibility weighted gradient echo MRI sequence (SWI) (TE/TR 20/28 ms; voxel size: 0.7 x 0.6 x 1.22 mm^3^), a fluid-attenuated inversion recovery sequence (FLAIR) (TR/TE/inversion time 4800/388/1800 ms; voxel size 0.49 x 0.49 x 1.00 mm^3^), and a 2D EPI pseudo-continuous arterial spin labeling (pCASL) sequence (voxel size 3.0 x 3.0 x 7.0 mm^3^; TR/TE 6000/14 ms, label duration 1650 ms, post labeling delay 1525-2275 ms, no background suppression). Presence of cortical, subcortical and lacunar infarcts was visually rated on the T1-weighted and FLAIR images by two experienced neuro-radiologists (JB and TW) according to the standards for reporting vascular changes on neuroimaging (STRIVE) criteria 18.

#### Quantification of WMH volume and WMH shape

Determination of WMH volume and WMH shape have previously been described 19, 20. In short, the FLAIR images were registered to the T1-weighted images using statistical parametric mapping version 12 (SPM12; Wellcome Institute of Neurology, University College London, UK, http://www.fil.ion.ucl.ac.uk/spm/doc/) for Matlab (The MathWorks, Inc., Natick, Massachusetts, United States). WMH segmentations were performed on the registered 3D FLAIR images by the lesion prediction algorithm (Schmidt, 2017, Chapter 6.1^44^) of the lesion segmentation toolbox version 2.0.15 (www.statistical-modeling.de/lst.html) for SPM12. For longitudinal (pre- to postoperative) WMH volume analysis, volumes were calculated from the probabilistic WMH segmentations. Segmentation of the lateral ventricles was performed on the T1-weighted images using the automated lateral ventricle delineation toolbox (ALVIN) for SPM8. To classify the WMH segmentations, probabilistic WMH segmentations were thresholded at 10%. WMH within 10 mm from the lateral ventricles into the white matter were considered periventricular WMH. WMH that extended from periventricular to more than 10 mm into the deep white matter were considered confluent WMH. WMH that were located >10 mm from the lateral ventricles were considered deep WMH. If present, cortical infarcts were manually delineated by a trained researcher (IK) and removed from the WMH segmentations. Baseline WMH volumes and WMH shape markers were calculated from the thresholded WMH segmentations 20. The WMH shape markers solidity, convexity, concavity index and fractal dimension of periventricular and confluent WMH were calculated by reconstruction of the convex hull, volume and surface area of all individual lesions. The WMH shape markers eccentricity and fractal dimension were calculated for deep WMH only (see 20). For all WMH shape markers, mean values per marker were calculated per patient and used for further analysis.

#### CBF quantification

ASL images were processed with ExploreASL 21. Lesion filling of the T1-weighted images was performed by the lesion segmentation toolbox version 2.0.15 (www.statistical-modeling.de/lst.html) for SPM12. The filled T1-weighted images were segmented using CAT12 (Gaser and Dahnke, Jena University Hospital, Departments of Psychiatry and Neurology, http://www.neuro.uni-jena.de/cat/index.html#About). CBF images were motion corrected and registered to the probabilistic gray matter volume maps. The CBF images were quantified with a single compartment model, after which the mean CBF was obtained for a total gray matter perfusion. All images were checked for image quality by a researcher (IK). Images that were classified as a good CBF contrast were used in the perfusion analysis. The no contrast images that contained noise only or large artifacts were excluded from further analysis.

### Statistical analysis

Baseline, intraoperative and clinical outcome data are expressed as median [25%, 75% quartiles], or frequencies (%), respectively. Differences in frequencies were tested by the Chi-Square-test. Differences between groups in terms of continuous parameters were tested by using non-parametric Mann-Whitney-U test for independent groups.

To assess the relation between preoperative cerebrovascular markers and intraoperative hyperglycemia, we performed multivariable logistic regression analysis adjusted for age and sex. The WMH shape marker analyses were also performed additionally adjusted for WMH volume, to assess whether the observed associations were independent of WMH volume. The regression analysis for WMH volume was additionally adjusted for total intracranial volume. We analyzed whether the occurrence of intraoperative hyperglycemia was associated with pre- to postoperative changes in WMH volume by linear regression analyses adjusted for age, sex, preoperative WMH volume and total intracranial volume. The association between intraoperative hyperglycemia and postoperative WMH volume at 3 months follow-up was studied by a linear regression model adjusted for age, sex and total intracranial volume at follow-up. In case of non-normally distributed data, all values were multiplied by 10,000 and then log transformed using the natural logarithm. IBM© SPSS© Statistics (version 25) was used for all analyses. A two-tailed P value ≤ 0.05 was considered to indicate statistical significance.

## Results

### Study population

Sixty-five patients were included into our analyses. Overall, 173 blood glucose levels were obtained (median 2 per patient; for details see 15). Glucose levels ranged between 73 mg/dL and 271 mg/dL with a median of 128 mg/dL (25%, 75% quartiles 109 mg/dL - 152 mg/dL). Eighteen patients (28%) showed intraoperative hyperglycemia (glucose maximum ≥ 150 mg/dL). Twenty-three patients (35%) were postoperatively admitted to the intensive care unit and the other 42 (65%) were transferred to a normal ward after the operation. Sixty-four patients received general anesthesia, whereas spinal anesthesia was applied in one patient. Patient characteristics, intraoperative parameters and outcome parameters are shown in Table 1 and also in 15. Patients who developed intraoperative hyperglycemia had a higher body mass index (BMI), preoperative blood glucose, as well as HbA1c levels and were more likely to have a preoperative diagnosis of diabetes compared to patients without intraoperative hyperglycemia (Table 1).

### The association between baseline cerebrovascular markers and intraoperative hyperglycemia

A preoperative more smooth shape of periventricular and confluent WMH was related to the occurrence of intraoperative hyperglycemia [convexity OR 33.318 (95 % CI (1.002 - 1107.950); p = 0.050] independent of age and sex. However, this association attenuated after additionally adjusting for WMH volume [OR 36.195 (95 % CI 0.939 - 1395.76); p = 0.054] (Supplemental Table S1), indicating that it is dependent on WMH volume. Other preoperative cerebrovascular markers were not related to the occurrence of intraoperative hyperglycemia (Table 2).

### The association of intraoperative hyperglycemia and pre- to postoperative changes in WMH volume and postoperative WMH volume

Due to large infarcts and MRI artefacts, only 41 patients could be included in the analyses of pre- to postoperative changes in WMH and postoperative WMH, respectively. Patients with intraoperative hyperglycemia showed no difference in pre- to postoperative change in WMH volume between baseline and the 3 month follow-up compared to patients without intraoperative hyperglycemia [B 0.556 (95% CI -0.479 - 1.590); p = 0.280; n=41] while adjusting for age, sex, baseline WMH volume and total intracranial volume. Three patients developed new cerebral infarcts at 3 months follow-up, two patients without intraoperative hyperglycemia and one with intraoperative hyperglycemia. As these numbers were too low, no comparative analyses were performed. Patients with intraoperative hyperglycemia showed no difference in postoperative WMH volume at the 3 month follow-up compared to patients without intraoperative hyperglycemia [B 0.105 (95% CI -0.147 - 0.356); p = 0.404; n=41].

## Discussion

We showed that a preoperative more smooth shape of periventricular and confluent WMH was related to the occurrence of intraoperative hyperglycemia. Other preoperative cerebrovascular markers were not related to the occurrence of intraoperative hyperglycemia. Intraoperative hyperglycemia showed no relation with pre- to postoperative changes in WMH volume nor with postoperative WMH volume at 3 months follow-up.

To the best of our knowledge, our study is the first to study the association between WMH shape markers and hyperglycemia in the perioperative context. Few previous studies have been performed that assessed the shape of WMH in relation to clinical outcomes. In one previous study, de Bresser et al. found higher eccentricity of deep WMH in a frontal and parietal location in patients with diabetes compared to healthy controls 22. In other studies it was shown that a more complex WMH shape was related to presence of lacunar infarcts in patients with symptomatic atherosclerotic disease, and to physical frailty in patients who were scheduled for major elective surgery 19, 23. Contrary to these previous studies, we found associations with a more smooth WMH shape. This suggests that WMH shape and the occurrence of intraoperative hyperglycemia might share a common pathway involving hypoperfusion and mechanisms related to small vessel disease. However, the underlying mechanisms related to a certain WMH shape are probably complex and in the case of intraoperative hyperglycemia is probably related to another mechanism compared to that in symptomatic artherosclerotic disease and frailty.

Our results point out the relevance of investigating cerebrovascular markers in identifying patients who are vulnerable to intraoperative and postoperative adverse outcomes, such as hyperglycemia 24. As early signs of cerebral damage can be detectable on MRI scans before it becomes clinically apparent, this method might be particularly valuable in early recognition of patients at risk for intraoperative and postoperative adverse outcomes.

The majority of previous studies on the influence of hyperglycemia on other cerebrovascular markers have only assessed patients with diabetes 8, 25, 26. Reitz et al. reported an association of diabetes-related hyperglycemia and a larger WMH volume and larger number of cerebral infarcts compared to control subjects 8. Patients with type 1 diabetes mellitus tend to develop WMH along with cognitive decline at a younger age than non-diabetic controls 27. Ogama et al. 28 found that postprandial hyperglycemia is related to WMH in diabetic patients with Alzheimer's disease while diabetic patients without dementia appeared to be less susceptible to the effects of hyperglycemia. Lacunar infarcts have been seen in patients with prediabetes and at an even higher number in patients with type 2 diabetes mellitus 29, 30.

Our findings might have clinical implications. There is evidence that hyperglycemia is associated with adverse clinical outcome, poor cognitive outcome in particular 1, 2, 4, 31, 32. In the future, cerebrovascular markers might be used to predict preoperative risk for intraoperative hyperglycemia. This might lead to patient-centered enhanced glycemic control for patients at risk. Furthermore, the optimal blood glucose target range in perioperative care remains subject of extensive research and of controversial debates as does the extent of glycemic control 33, 34. To balance prevention from hyperglycemia-associated complications with the risk of fatal hypoglycemia, constitutes a challenge in perioperative care and outside the surgical population alike.

The strengths of our study are the prospectively collected MRI data including a follow-up at 3 months as well as the advanced assessment of WMH volume and shapes. Some limitations of the present study need to be mentioned. First, our study is limited by a relatively small cohort including only patients aged ≥ 65 years. Second, part of the data including intraoperative blood glucose measurements was obtained retrospectively, bearing the risk of missed patients who had hyperglycemia. Third, patients with intraoperative blood glucose measurement were potentially undergoing more extended surgery with a higher risk to develop intraoperative hyperglycemia or had more severe underlying conditions warranting intensive glycemic control and thus creating a potential selection bias in our dataset.

In conclusion, our study is the first to investigate the association between cerebrovascular markers and intraoperative hyperglycemia. We found that a preoperative more smooth shape of periventricular and confluent WMH was related to the occurrence of intraoperative hyperglycemia. These findings may suggest that a similar underlying mechanism leads to a certain pattern of vascular brain abnormalities and an increased risk of hyperglycemia.

## Supplementary Material

Supplementary table S1.

## Figures and Tables

**Table 1:** Patient characteristics, intraoperative parameters and outcome parameters.

	Total group(n=65)	Patients with intraoperative glucose < 150 mg/dL(n=47)	Patients with intraoperative Glucose ≥ 150 mg/dL(n=18)	P value (< 150 vs. ≥ 150)
**Preoperative patient characteristics**				
Age [years]	72 (68-75)	71 (68-75)	72 (68-75)	0.740
Male sex [n] (%)	46 (71 %)	31 (66 %)	15 (83 %)	0.229
Body Mass Index [kg/m²]	25.8 (24.0-29.1)	25.4 (23.6-27.3)	28.2 (26.2-30.6)	**0.002**
ASA score II/III	40 (62 %) / 25 (38 %)	31 (66 %) / 16 (34 %)	9 (50 %) /9 (50 %)	0.266
Baseline MMSE	29 (28-30)	29 (28-30)	29 (29-30)	0.673
Diabetes in history [n] (%)	27 (42 %)	11 (23 %)	16 (89 %)	**<0.001**
Preoperative glucose levels [mg/dL]	101 (90-120)	96 (86-107)	132 (115-180)	**<0.001**
Preoperative HbA1c [%]	5.9 (5.5-6.7)	5.7 (5.4-6.0)	7.2 (6.7-7.7)	**<0.001**
**Intraoperative parameters**				
Surgical time [min]	147 (100-219)	155 (100-222)	132 (115-180)	0.747
Intra-abdominal or intra-thoracic surgery [n] (%)	37 (57 %)	29 (62 %)	8 (44 %)	0.267
Intraoperative glucose minimum [mg/dL]	114 (103-135)	107 (98-119)	154 (135-178)	**<0.001**
Intraoperative glucose mean [mg/dL]	120 (109-147)	113 (104-124)	167 (154-183)	**<0.001**
Intraoperative glucose maximum [mg/dL]	125 (111-154)	117 (106-131)	181 (171-203)	**<0.001**
**Outcome parameters**				
Hospital duration [days]	8 (5-13)	8 (5-14)	7 (6-10)	0.843
Patients with postoperative infections* [n] (%)	6 (9 %)	5 (11 %)	1 (6 %)	0.670

Continuous quantities in median with quartiles; ASA, *American Society of Anesthesiologists;* BG, *blood glucose;* HbA1c, *glycated hemoglobin;* MMSE, *Mini Mental Status Examination.* *These included 3 patients with cholangitides, 1 patient with a pneumonia, 1 patient with a meningitis, and 1 patient with a wound infection. P values were calculated using the Mann-Whitney-U test or the χ2 test for continuous and categorical variables, respectively. Of note, this Table has been reported previously 15.

**Table 2:** The association between preoperative cerebrovascular markers and occurrence of intraoperative hyperglycemia.

	Total group	Patients with intraoperative glucose <150 mg/dL	Patients with intraoperative glucose ≥150 mg/dL	OR (95 % CI)	P value
WMH volume (n = 59: 42 versus 17)*					
Total WMH volume, mL	2.1 (0.8-4.3)	1.87 (0.62-4.18)	2.43 (0.95-4.67)	1.506 (0.814 - 2.785)	0.192
Periventricular/confluent WMH volume, mL	2.1 (0.8-4.2)	1.9 (0.6-4.0)	2.3 (0.9-4.6)	1.471 (0.805 - 2.689)	0.210
Deep WMH volume, mL	0.04 (0.00-0.14)	0.02 (0.00-0.10)	0.08 (0.00-0.15)	0.973 (0.539 - 1.756)	0.926
Periventricular/confluent WMH shape (n = 59: 42 versus 17)*					
Fractal dimension	1.54 (1.31-1.69)	1.54 (1.29-1.66)	1.60 (1.39-1.72)	0.919 (0.003 - 245.532)	0.976
Solidity	0.33 (0.25-0.62)	0.39 (0.26-0.63)	0.31 (0.22-0.51)	0.434 (0.121 - 1.555)	0.200
Convexity	1.15 (1.01-1.29)	1.14 (1.01-1.26)	1.26 (1.05-1.43)	33.318 (1.002 - 1107.950)	0.050
Concavity index	1.03 (1.00-1.09)	1.03 (1.00-1.09)	1.03 (0.97-1.08)	0.007 (0.000 - 10.792)	0.186
Deep WMH shape (n = 39: 26 versus 13)*					
Fractal dimension	1.82 (1.68-2.01)	1.81 (1.67-1.99)	1.89 (1.68-2.04)	1.226 (0.127 - 11.835)	0.860
Eccentricity	0.54 (0.46-0.59)	0.56 (0.46-0.60)	0.52 (0.45-0.57)	0.338 (0.018 - 6.252)	0.466
Cerebral infarcts (n = 61: 43 versus 18)*					
Patients with lacunar infarcts, n (%)	20 (33 %)	13 (30 %)	7 (39 %)	1.283 (0.379 - 4.345)	0.688
Patients with subcortical infarcts, n (%)	1 (2 %)	1 (2 %)	0 (0 %)	1.283 (0.379 - 4.345)	0.688
Patients with cortical infarcts, n (%)	13 (22 %)	9 (21 %)	4 (22 %)	1.101 (0.284 - 4.260)	0.889
Perfusion (n = 40: 29 versus 11)*					
Gray matter perfusion	68.1 (55.5-87.3)	65.6 (54.9-83.4)	68.4 (62.9-88.3)	1.000 (0.975 - 1.025)	0.997

Associations between preoperative cerebrovascular markers and intraoperative hyperglycemia assessed by multiple logistic regression analyses adjusted for age and sex. WMH volumes were additionally adjusted for intracranial volume. Continuous quantities are shown as medians with quartiles. *Due to neurosurgical interventions, oversegmentation, large infarcts, artefacts or absence of certain markers (for example deep WMH), the respective parameters had to be excluded from analysis. Due to non-normal distribution, WMH volume markers, WMH volume change, solidity, and eccentricity were multiplied by 10,000 and then natural log transformed. *WMH: white matter hyperintensities.*
