# Supplementary material for: The association between intraoperative hyperglycemia and cerebrovascular markers
Source: Int J Med Sci. 2021 Jan 21;18(6):1332–8. doi: 10.7150/ijms.51364 (PMC7893564; doi:10.7150/ijms.51364)
Supplement: Supplementary file 1 — Supplementary table S1. [file ijmsv18p1332s1.pdf]

Supplement

**Tabel S1: The association between preoperative WMH shape markers and occurrence of intraoperative hyperglycemia; analyses additionally adjusted for WMH volume.**

| | Total group (n=65) | Patients with intraoperative glucose <150 mg/dL (n=47) | Patients with intraoperative glucose ≥150 mg/dL (n=18) | OR (95 % CI) | P value |
|---|---|---|---|---|---|
| **Periventricular/confluent WMH shape** | | | | | |
| Fractal dimension | 1.54 (1.31-1.69) | 1.54 (1.29-1.66) | 1.60 (1.39-1.72) | 1.170 (0.016 – 85.788) | 0.943 |
| Solidity | 0.33 (0.25-0.62) | 0.39 (0.26-0.63) | 0.31 (0.22-0.51) | 0.198 (0.026 – 1.511) | 0.118 |
| Convexity | 1.15 (1.01-1.29) | 1.14 (1.01-1.26) | 1.26 (1.05-1.43) | 36.195 (0.939 – 1395.76) | 0.054 |
| Concavity index | 1.03 (1.00-1.09) | 1.03 (1.00-1.09) | 1.03 (0.97-1.08) | 0.001 (0.000 – 3.593) | 0.097 |
| **Deep WMH shape** | | | | | |
| Fractal dimension | 1.82 (1.68-2.01) | 1.81 (1.67-1.99) | 1.89 (1.68-2.04) | 1.170 (0.016 – 85.788) | 0.943 |
| Eccentricity | 0.54 (0.46-0.59) | 0.56 (0.46-0.60) | 0.52 (0.45-0.57) | 0.293 (0.016 – 5.303) | 0.406 |

Associations between preoperative WMH shape markers and occurrence of intraoperative hyperglycemia assessed by multiple logistic regression analyses adjusted for age, sex and WMH volume.
*WMH: white matter hyperintensities.*
